# 725 Evaluating Wound Care Compliance, Healing and Perceptions in Burn Patients Discharged to Post-Acute Care Facilities

**DOI:** 10.1093/jbcr/irad045.200

**Published:** 2023-05-15

**Authors:** Suzanne Osborn, Kristina Garn, Griselda Mitchell, Joya Neal, Juan Flores, Karen Richey, Kevin Foster

**Affiliations:** AZ Burn Center at Valleywise Health, Phoenix, Arizona; Arizona Burn Center at Valleywise Health, Phoenix, Arizona; Arizona Burn Center at Valleywise Health, Phoenix, Arizona; Arizona Burn Center @ Valleywise Health Medical Center, Gilbert, Arizona; AZ Burn Center at Valleywise Health, Scottsdale, Arizona; Arizona Burn Center Valleywise Health, Phoenix, Arizona; AZ Burn Center at Valleywise Health, Phoenix, Arizona; AZ Burn Center at Valleywise Health, Phoenix, Arizona; Arizona Burn Center at Valleywise Health, Phoenix, Arizona; Arizona Burn Center at Valleywise Health, Phoenix, Arizona; Arizona Burn Center @ Valleywise Health Medical Center, Gilbert, Arizona; AZ Burn Center at Valleywise Health, Scottsdale, Arizona; Arizona Burn Center Valleywise Health, Phoenix, Arizona; AZ Burn Center at Valleywise Health, Phoenix, Arizona; AZ Burn Center at Valleywise Health, Phoenix, Arizona; Arizona Burn Center at Valleywise Health, Phoenix, Arizona; Arizona Burn Center at Valleywise Health, Phoenix, Arizona; Arizona Burn Center @ Valleywise Health Medical Center, Gilbert, Arizona; AZ Burn Center at Valleywise Health, Scottsdale, Arizona; Arizona Burn Center Valleywise Health, Phoenix, Arizona; AZ Burn Center at Valleywise Health, Phoenix, Arizona; AZ Burn Center at Valleywise Health, Phoenix, Arizona; Arizona Burn Center at Valleywise Health, Phoenix, Arizona; Arizona Burn Center at Valleywise Health, Phoenix, Arizona; Arizona Burn Center @ Valleywise Health Medical Center, Gilbert, Arizona; AZ Burn Center at Valleywise Health, Scottsdale, Arizona; Arizona Burn Center Valleywise Health, Phoenix, Arizona; AZ Burn Center at Valleywise Health, Phoenix, Arizona; AZ Burn Center at Valleywise Health, Phoenix, Arizona; Arizona Burn Center at Valleywise Health, Phoenix, Arizona; Arizona Burn Center at Valleywise Health, Phoenix, Arizona; Arizona Burn Center @ Valleywise Health Medical Center, Gilbert, Arizona; AZ Burn Center at Valleywise Health, Scottsdale, Arizona; Arizona Burn Center Valleywise Health, Phoenix, Arizona; AZ Burn Center at Valleywise Health, Phoenix, Arizona; AZ Burn Center at Valleywise Health, Phoenix, Arizona; Arizona Burn Center at Valleywise Health, Phoenix, Arizona; Arizona Burn Center at Valleywise Health, Phoenix, Arizona; Arizona Burn Center @ Valleywise Health Medical Center, Gilbert, Arizona; AZ Burn Center at Valleywise Health, Scottsdale, Arizona; Arizona Burn Center Valleywise Health, Phoenix, Arizona; AZ Burn Center at Valleywise Health, Phoenix, Arizona

## Abstract

**Introduction:**

Prior research at our center demonstrated that 51% of all patients discharged to a skilled nursing facility (SNF) or acute rehabilitation center (ARC) had deterioration of their wounds. In that review, we were unable to identify specific patient factors associated with a worsening of wounds. We postulated that there was a relationship between specific facilities and outcomes, however due to the number of discharge facilities we were unable to analyze those relationships. Following our analysis, clinic personnel increased interactions with facilities to improve follow-up and communication. The purpose of this quality improvement project was to further explore factors that may influence wound healing in SNF and ARC environments and improve patient outcomes.

**Methods:**

The review occurred over a 3.5-month period. Burn and non-burn patients residing at a SNF or ARC at the time of their clinic visit were included in the sample. A 10-item survey was administered to patients and a 5-item survey to the clinician. Wounds overall were deemed as “Improved” (IM) or “Worsened/No Change” (W/NC) and patients subsequently divided into these cohorts for analysis. Basic statistics were calculated.

**Results:**

A total of 55 patients completed 81 surveys. The majority, 69% (n=38) had wounds that improved and 31% (n=17) showed worsening or no change. There were no significant differences between groups in wound etiology, facility type, dressing type / frequency or the absolute number of dressing regimes/patient. Facility compliance with clinic dressing orders was mediocre with 65% compliance in IM and 57% in the W/NC (p=.484). When asked if wounds were progressing as expected clinicians responded yes 97% of the time for IM and 36% for W/NC (p< .00001). When patients were asked if their facility had the needed dressing supplies, 84% (n=46) IM responded yes vs 61% W/NC (p=.029). There were no significant differences in patient understanding of wound care, if performed correctly, or ratings of pain control, appetite, restfulness, staff responsiveness, and overall care at the SNF or ARC.

**Conclusions:**

An improvement in outcomes was noted between our first analysis and the current evaluation, this may be related to increased communication with facilities. Although the data demonstrated that prescribed wound care was not adhered to, there was no difference between groups. However, patient reports of inadequate dressing supplies at the facilities may be a factor in the progression of wound healing. While overall outcomes were improved, patients continue to experience an unacceptable rate of wound deterioration and further investigation is warranted.

**Applicability of Research to Practice:**

Identifying interventions for patients sent to SNF and ARC facilities would improve wound outcomes.

